# An investigation of the growth status of 19-year-old *Idesia polycarpa* ‘Yuji’ plantation forest in the mountainous region of Henan, China

**DOI:** 10.1016/j.heliyon.2023.e19716

**Published:** 2023-09-01

**Authors:** Pengcheng Li, Sohel Rana, Mengxing Zhang, Chao Jin, Kaixin Tian, Zhen Liu, Zhi Li, Qifei Cai, Xiaodong Geng, Yanmei Wang

**Affiliations:** College of Forestry, Henan Agricultural University, Zhengzhou, 450046, China

**Keywords:** *Idesia polycarpa* ‘Yuji’, Forests plantation, Soil nutrients, Forest stand investigation, Fruit oil content

## Abstract

Plantation forests play an important role in the mitigation of greenhouse gas emissions. *Idesia polycarpa* Maxim is an emerging woody oil tree species in most Asian countries. The 19-year-old *Idesia polycarpa* ‘Yuji’ plantation forest was selected as a sample site. The nutrient contents of the understory soil total carbon (TC), total nitrogen (TN), nitrate nitrogen (NN), organic carbon (OC), available phosphorus (AP), available potassium (AK), and pH were analyzed. Several metrics were measured to quantify the growth status of the forest, such as tree heights (H), clear bole heights (CBH), diameters at breast height (DBH), and male-to-female ratios (MFR). In addition, we harvested the fruits to analyze oil content and fatty acid composition. The results found that the nutrient content of the soil was TC (4.93%), TN (0.42%), NN (43.08 mg kg^−1^), OC (4.90 g kg^−1^), AP (13.66 mg kg^−1^), AK (30.48 mg kg^−1^), and pH (7.90). The growth characteristics were H (11.75 m), DBH (12.79 cm), and CBH (6.17 m). The MFR was close to 1:1. Besides, the oil content of the fruit and unsaturated fatty acids was 24.08% and 68.49%, respectively. As an alternative tree species, the plantation of *Idesia polycarpa* offers great potential in artificial afforestation in some particular places with specific forest site conditions.

## 1 Introduction

Forest plays a vital role in reducing pressure on the natural environment, weakening the greenhouse effect, and storing carbon dioxide (CO_2_) in the atmosphere [1,2]. Using carbon sinks reduces the burden caused by greenhouse gas emissions [3]. Afforestation can enhance the carbon sink capacity of forests, which is an effective way to increase carbon stocks [1]. Establishing plantation forests can mitigate climate change, restore degraded land, and maintain ecosystem functions and services (e.g., provision of water resources, increased biodiversity, recreational functions, etc.) [4]. It is widely used in restoring disturbed ecosystems, combating desertification and soil erosion [5]. Expanding the cover area of artificial forests is one of the effective ways to solve the problem of the rapid decrease of natural forest area. Planted forests greatly supply wood products, conserve water and soil, and prevent wind [6]. Besides, artificial forests can produce a large amount of biomass (e.g., wood, branches, leaves, fruits, extracts, etc.) in a short time. Biomass is a renewable energy source that can be used to produce heat and electricity [7]. Successful afforestation strategies can provide remarkable ecological and economic value [8]. As the world's largest planted forest area, planted forests play a vital role in the preservation of the ecological environment and the development of the economy in China [9].

Soil is an essential component of terrestrial ecosystems, a significant source of nutrients for plant growth and development [10], and the basis for supporting forest growth and development [11]. Soil nutrient availability is one of the environmental and biological drivers of plantation productivity [12]. Forest productivity is closely related to soil, and the decline in productivity may be related to the reduction of soil's organic matter and nutrient content [13]. In artificial forests, plant communities interact with the soil. On the one hand, plant communities can accumulate organic matter and may alter soil nutrient availability and water content [14]. Plants can also fix carbon and nitrogen from the atmosphere and input them into the soil subsystem through litter and root exudates [15]. On the other hand, soil's physicochemical properties may affect plants' composition and structure [14]. For example, soil pH affects many biological and physical properties and processes influencing plant growth and biomass production [16]. Batjes [17] reported the importance of soil total Carbon and Nitrogen and their relationship to global climate change. Organic matter positively affects soil aggregation [18] and is the primary determinant of soil carbon storage and nutrient mineralization rates [19]. Besides, nutrients such as Nitrogen, Phosphorus, and Potassium stored in the soil play a key role in plant growth and determine the ecosystem's structure, function, and productivity level [20,21].

*Idesia polycarpa* Maxim is a deciduous tree belonging to the family of Flacourtiaceae *and* a positive fast-growing tree species. It has strong resistance and wide adaptability [22]. *It* takes around 4 or 5 years to reach reproductive maturity [23,24]. Both fruits and seeds of *Idesia polycarpa* can extract oil [25], its oil has many advantages [26,27], andrenowned as ‘Beautiful Tree Oil Depot’ and ‘Chinese Olive Oil’ [28]. Even the oil residue performs well in high-efficient energy storage [29] and can also be used as excellent garden tree species [30,31], timber forest, and economic forest tree [32]. Therefore, *Idesia polycarpa* has attracted significant attention from researchers [[33], [34], [35], [36]].

However, it is still unclear whether *Idesia polycarpa* can be used as a tree species for afforestation and whether it can grow normally under challenging site conditions. Thus, in this study, the soil nutrient content of *Idesia polycarpa* ‘Yuji’ forest was measured, the growth status of the stand under such a mountainous soil environment was investigated, and the fatty acid composition of the fruit was preliminarily analyzed. We believe better promoting and utilizing this species (*Idesia polycarpa* ‘Yuji’) will provide a new choice for afforestation under challenging conditions.

## 2 Materials and methods

### 2.1 Study site

**Fig. 1:**
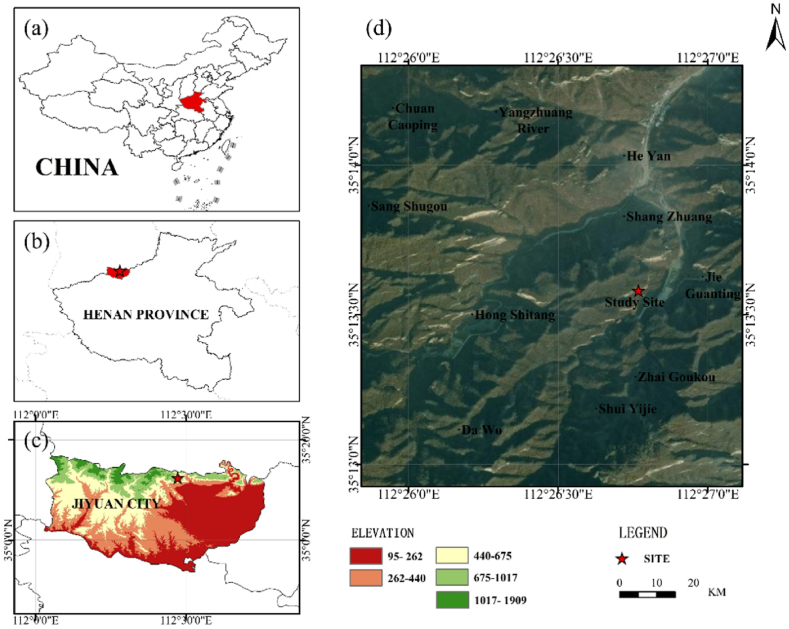
Overview of the study site. (a) Map of the People's Republic of China, (b) map of the Henan Province, (c) map of Jiyuan city, (d) the map of the sample collection point identification by star (☆) mark.

The experimental area is located in the Jiuligou forest area of Jiyuan Yugong Forest Farm (112°29′47″E and 35°13′33″N), around 40 km northwest of Jiyuan City, Henan Province (Fig. 1). It is a mid-temperate continental monsoon-type climate with four distinct seasons: Spring (dry with little rain), Summer (rainy and humid), Autumn (high and cool), and Winter (dry and cold). The annual average temperature is 14.1 °C (minimum −20 °C -maximum 43.4 °C). The average frost-free period is 223 days, the average annual sunshine hours are 2375.4 h, the sunshine rate is 54%, and the average annual precipitation is 650 mm.

### 2.2 Data sampling and preparation

In 2004, 2-year-old seedlings of *Idesia polycarpa* ‘Yuji’ (‘Yuji’ was recognized as a new variety of *Idesia polycarpa* by the Forest Tree Species Validation Committee of Henan Province in 2014, certified as a forest tree species in 2020.) were planted in Yugong Forest Farm, with row spacing of 3 m × 5 m.

The samplings were conducted at three (650 m, 690 m, and 720 m) different elevations of the study site. Three sampling points were randomly selected at each altitude, with a total of 9 sampling points. At each sampling point, soil samples were collected at depths of 0–10, 10–20, 20–30, 30–40, and 40–50 cm. A sample of 500 g was collected from each sampling point with a total of 45 samples. Soil samples were collected from an excavation of the soil profile (Fig. S1). The samples were separately stored and labeled to avoid contamination.

Soil pH was determined using the potentiometric method (2.5:1) [37]. Total carbon (TC) and total nitrogen (TN) were determined using the automatic elemental analyzer (EURO EA3000, Italy) [38,39]. The contents of nitrate nitrogen (NN) and organic carbon (OC) were systematically studied using soil nutrient status (ASI method) [40,41]. The sodium bicarbonate extraction-molybdenum antimony colorimetric method was used to determine the available phosphorus (AP) content [42]. The available potassium (AK) content was determined using the soil nutrient rapid analyzer (TPY-8A, China) [43,44].

The tree species composition and growth status ‘Yuji’ were investigated. Tree height (H), clear bole height (CBH), and diameter at breast height (DBH) in the forest were measured using the measuring tape, and the proportion of male and female trees was calculated. We randomly selected 109 trees at different altitudes (650 m, 690 m, and 720 m).

The ‘Yuji’ fruits were collected to determine the oil content and fatty acids. Oil content in fruits was determined using Soxhlet extraction [45,46], and the potassium hydroxide/methanol method was used to determine the composition and content of fatty acids in oil [47].

### 2.3 Statistical analysis

All statistical analyses were performed using the IBM SPSS Statistics v. 26 (IBM Corp., Armonk, NY, USA) program. We analyzed the soil nutrient content using One-way ANOVA and LSD to identify the significant differences between elevations and soil depths. The data results of the fatty acids content and the Plant H, CBH, and DBH were summarized using Microsoft Excel v. 2016 (Microsoft Corp., Redmond, WA, USA). The mapping software was ArcGIS 8.0 (ESRI, USA).

## 3 Results

### 3.1 Analysis of soil nutrient indexes under Idesia polycarpa ‘Yuji’ forest

According to the experimentation results of 45 soil samples collected in 2020, the nutrient indexes of the soil under the ‘Yuji’ forest at different altitudes are shown in Table 1. The soil under the ‘Yuji’ forest was relatively barren; most were stone except for leaf litter (Fig. S1). At the elevation of 690–720 m, the average soil pH (7.90 ± 0.01), TC (4.93 ± 0.14%), TN (0.42 ± 0.01%), NN (43.08 ± 1.06 mg kg^−1^), OC (4.90 ± 0.15 g kg^−1^) AP (13.66 ± 0.13 mg kg^-1-1^), and AK (30.48 ± 1.28 mg kg^−1^) was detected.

**Table 1:** Table of basic nutrient indexes of soil under *I. polycarpa* ‘Yuji’ forest.

Altitude (m)	Soil depth (cm)	pH (2.5:1)	TC (%)	TN (%)	NN (mg kg^−1^)	OC (g kg^−1^)	AP (mg kg^−1^)	AK (mg kg^−1^)
650	0–10	7.93 ± 0.03^Aa^	5.73 ± 0.60^Aa^	0.44 ± 0.03^Ab^	37.28 ± 3.70^Ab^	7.20 ± 0.19^Aa^	16.07 ± 0.80^Aa^	50.11 ± 0.94^Aa^
	10–20	7.92 ± 0.01^Aa^	5.37 ± 0.71^Aa^	0.40 ± 0.02^ABb^	33.86 ± 4.60^Ab^	7.03 ± 0.10^Aa^	14.19 ± 0.34^Aa^	52.44 ± 1.85^Aa^
	20–30	7.93 ± 0.02^Aa^	5.57 ± 0.52^Aa^	0.38 ± 0.03^ABb^	36.17 ± 5.66^Ab^	7.41 ± 0.49^Aa^	14.25 ± 0.52^Aa^	40.20 ± 3.29^Ba^
	30–40	7.91 ± 0.02^Aab^	5.32 ± 0.66^Aa^	0.37 ± 0.03^ABb^	32.31 ± 5.61^Ab^	6.93 ± 0.42^Aa^	14.32 ± 0.49^Aa^	28.90 ± 3.81^Ca^
	40–50	7.93 ± 0.01^Aa^	5.30 ± 0.54^Aa^	0.35 ± 0.03^Bb^	32.07 ± 6.11^Ab^	6.44 ± 0.39^Aa^	14.51 ± 0.74^Aa^	30.40 ± 2.40^Ca^
690	0–10	7.87 ± 0.02^ABa^	4.00 ± 0.28^Ab^	0.38 ± 0.01^Ab^	44.80 ± 2.08^Aab^	4.36 ± 0.07^Ab^	14.10 ± 0.39^Ab^	39.96 ± 10.62^Aa^
	10–20	7.85 ± 0.02^Bb^	3.63 ± 0.38^Ab^	0.36 ± 0.04^Ab^	44.88 ± 1.17^Aa^	4.53 ± 0.06^Ab^	13.78 ± 0.52^Aa^	24.89 ± 2.57^Bb^
	20–30	7.89 ± 0.02^ABa^	3.89 ± 0.13^Ab^	0.34 ± 0.02^Ab^	44.64 ± 2.65^Aab^	3.96 ± 0.04^Bb^	12.87 ± 0.41^ABa^	24.31 ± 3.46^Bb^
	30–40	7.94 ± 0.03^Aa^	3.30 ± 0.49^Ab^	0.35 ± 0.04^Ab^	49.38 ± 1.92^Aa^	4.64 ± 0.20^Ab^	12.26 ± 0.48^Bb^	22.49 ± 3.44^Ba^
	40–50	7.92 ± 0.03^ABa^	3.27 ± 0.20^Ab^	0.37 ± 0.05^Aab^	46.44 ± 2.58^Aa^	4.45 ± 0.13^Ab^	12.26 ± 0.29^Bb^	16.00 ± 2.53^Bb^
720	0–10	7.90 ± 0.03^Aa^	5.56 ± 0.25^Aa^	0.54 ± 0.03^Aa^	52.05 ± 1.53^Aa^	3.19 ± 0.18^Ac^	14.16 ± 0.31^Ab^	45.07 ± 3.85^Aa^
	10–20	7.89 ± 0.01^Aab^	5.42 ± 0.22^Aa^	0.52 ± 0.02^Aa^	47.87 ± 1.93^Aa^	3.24 ± 0.12^Ac^	13.67 ± 0.33^Aa^	32.46 ± 2.74^Bb^
	20–30	7.89 ± 0.02^Aa^	5.80 ± 0.33^Aa^	0.48 ± 0.01^Aa^	51.21 ± 3.02^Aa^	3.35 ± 0.13^Ab^	13.86 ± 0.37^Aa^	27.55 ± 2.22^Bb^
	30–40	7.86 ± 0.03^Ab^	5.65 ± 0.25^Aa^	0.49 ± 0.02^Aa^	50.06 ± 3.72^Aa^	3.32 ± 0.15^Ac^	13.80 ± 0.49^Aa^	27.22 ± 1.62^Ba^
	40–50	7.90 ± 0.04^Aa^	6.23 ± 0.31^Aa^	0.48 ± 0.04^Aa^	43.17 ± 3.19^Aab^	3.26 ± 0.14^Ac^	13.62 ± 0.41^Aa^	18.60 ± 1.83^Cb^

Note: Different capital letters (ABC) indicate significant differences between different soil depths at the same altitude (P < 0.05), different lowercase letters (abc) indicate significant differences between different altitudes at the same soil depth (P < 0.05). Data values represent the mean ± SD. TC: soil total carbon content. TN: soil total nitrogen content. NN: soil nitrate nitrogen content. OC: soil organic carbon content. AP: soil available phosphorus content. AK: soil available potassium content.

Furthermore, at the same altitude, there was no significant difference in the contents of TC, NN, and OC among different soil depths (*P* > 0.05), and there was a significant difference in the content of AK (*P* < 0.05). At the same soil depth, TC, TN, NN, OC, AP, and AK contents significantly differed at different altitudes (*P* < 0.05). In summary, the nutrient indexes of soil under *Idesia polycarpa* ‘Yuji’ forest with different altitudes and soil depths were different.

### 3.2 The investigation status of Idesia polycarpa ‘Yuji’ plantation forest

#### 3.2.1 Tree description

The ‘Yuji’ crown was open, and the trunk was grayish-white and smooth. The leaf margin was rounded serrate, the leaf base was heart-shaped, palmately basal veins 5–7, and the petiole was reddish or green. The leaves were broadly ovate to ovate-centered, apex acute to acuminate (Fig. S2A). The abaxial leaf surface was pink, and the leaf veins had a few pubescences. The flowers were yellow and aromatic, the berries were spherical and red, and the fruit contained many small plump black seeds (Fig. S2B -2D).

#### 3.2.2 Stand growth

In the investigated ‘Yuji’ plantation forest, there were 58 males and 51 females, and the ratio of males to females was close to 1:1. The individual growth of trees was satisfactory, there were no obvious pests and diseases, and the stand density was 667 trees·ha^−1^. The canopy density was high, up to 0.8. The average H, CBH, and DBH were 11.75 ± 0.24 m, 6.17 ± 0.21 m, and 12.79 ± 0.48 cm, respectively (Table 2).

**Table 2:** The growth condition of *I. polycarpa* ‘Yuji’ forest. Data values represent the mean ± SD.

Statistic	Male	Female	Total
H (m)	11.50 ± 0.34	12.03 ± 0.32	11.75 ± 0.24
CBH (m)	6.01 ± 0.30	6.36 ± 0.30	6.17 ± 0.21
DBH (cm)	12.47 ± 0.71	13.15 ± 0.63	12.79 ± 0.48

Note: H: tree height. CBH: clear bole height. DBH: diameter at breast height.

#### 3.2.3 Tree species composition

The tree species composition of ‘Yuji’ forest is shown in Table 3. In the *Idesia polycarpa* ‘Yuji’ forest, a few other tree species are occasionally seen, and herb species are abundant under the forest. In addition, the Chinese herbs such as *Forsythia suspensa*, *Corydalis yanhusuo*, *Epimedium brevicornu*, and *Polygonatum sibiricum* grew well under the ‘Yuji’ forest, which also provided a potential direction for the future management of *Idesia polycarpa* forest.

**Table 3:** Species composition of *I. polycarpa* ‘Yuji’ forest.

Type	No.	Scientific name
Arbor	1	*Cornus officinalis*
	2	*Juniperus formosana*
	3	*Taxus wallichiana* var *chinensis*
	4	*Ulmus pumila*
	5	*Yulania denudata*
Shrub	1	*Acer tataricum* subsp. *ginnala*
	2	*Forsythia suspensa*
	3	*Lonicera japonica*
	4	*Pistacia chinensis*
Herb	1	*Aquilegia viridiflora*
	2	*Begonia fimbristipula*
	3	*Corydalis yanhusuo*
	4	*Dracocephalum rupestre*
	5	*Epimedium brevicornu*
	6	*Geranium wilfordii*
	7	*Iris lactea*
	8	*Lagopsis supina*
	9	*Ophiopogon bodinieri*
	10	*Phlomis umbrosa*
	11	*Polygonatum sibiricum*
	12	*Trachelospermum jasminoides*

#### 3.2.4 Fruit

The moisture content of the fruit was 35.98 ± 1.37%, and the oil content was 24.08 ± 0.60%. The top three unsaturated fatty acids in fruit oil were linoleic acid, oleic acid, and palmitoleic acid, and the saturated fatty acids were palmitic acid, tridecanoic acid, and stearic acid (Table 4). Among them, the linoleic acid content in fruit was up to 54.87 ± 1.82%. The extracted fruit oil of ‘Yuji’ is shown (Fig. S3).

**Table 4:** Composition and content of fatty acids in fruit oil of *I. polycarpa* ‘Yuji’ (%). Data values represent the mean ± SD.

Fatty acids	Fruit oil
Palmitoleic acid	4.22 ± 0.63
Oleic acid	8.49 ± 0.53
Linoleic acid	54.87 ± 1.82
α-Linoleic acid	0.83 ± 0.01
γ-Linoleic acid	0.08 ± 0.07
Unsaturated fatty acids	68.49 ± 2.28
Tridecanoic acid	4.09 ± 0.08
Myristic acid	0.17 ± 0.01
Palmitic acid	22.81 ± 0.90
Stearic acid	1.26 ± 0.04
Eicosanoic acid	0.28 ± 0.01
Saturated fatty acids	28.61 ± 0.98
Others	2.90 ± 0.13

## 4 Discussion

In 2020, the *Idesia polycarpa* ‘Yuji’ forest age was about 19 years, and this forest stand is an excellent area for studying *Idesia polycarpa species*. The stand grows in Yugong Forest Farm, located in Taihang Mountains. The Taihang Mountains are a significant ecological barrier within China, which belongs to a typical soil-rock mountain area in northern China [48]. Under the unique geological background and human activities, vegetation damage and soil erosion are severe in this area, which seriously threatens the safety of the North China Plain [49]. The importance of carrying out afforestation activities in this area cannot be overstated. However, due to the high content of soil gravel, barren soil layer, and discontinuous distribution in the Taihang Mountains, it is not easy to control the ecological environment [50].

In the context of the increasing demand for carbon dioxide sequestration in the atmosphere, it is crucial to sustainably maintain the soil fertility of plantation forests [51]. A recent study [52] measured the soil nutrients under the artificial forest of *Pinus tabuliformis*, and the contents of soil pH (8.4), total N (1.25 g kg^−1^), organic carbon (13.04 g kg^−1^) and available P (2.84 mg kg^−1^) were measured. The study found that the contents of organic carbon, available P, and total N in the soil of the plantation were significantly higher than those of the natural forest at the same density. In addition, the stand density and vegetation type significantly affected the soil properties. Similarly, an investigation conducted by Ref. [53] on the *Pinus massoniana* plantation (at 300 m altitude and 13 years old) found that the average DBH was 10.7 cm, and the plant height was 8.1 m. At the same time, the content of the soil pH (4.48), available P (1.89 mg kg^−1^), total N (1.68 g kg^−1^), and organic carbon (31.9 g kg^−1^) were reported. Besides, In the *Erythrophleum fordii* plantation (at 300 m altitude and 13 years old), the mean DBH (7.8 cm), tree height (8.0 m), soil pH (4.28), available P (1.96 mg kg^−1^), total N (1.65 g kg^−1^) and organic carbon (27.8 g kg^−1^) were found [53]. In this study, the stand status and soil nutrient content of *Idesia polycarpa* ‘Yuji’ plantation forest differed from the above stands. Which may be related to the type of stands (tree species and age of stand), geographical conditions (climate and altitude), and history of plantation uses of the study site.

The combination with soil system classification [[54], [55], [56]] and soil nutrient classification criteria [57], the average pH of the soil under the *Idesia polycarpa* ‘Yuji’ plantation forest was 7.90, belonging to neutral to slightly alkaline soil. In addition, the TC (4.93%), TN (0.42%, level 6), NN (43.08 mg kg^−1^), and OC (4.90 g kg-1, level 6) were found, which was only a quarter of the average soil organic matter content in Henan Province [58]. The contents of AP and AK were 13.66 mg kg^−1^ (level 3), and 30.48 mg kg^−1^ (level 5), respectively. Plantation forest soil at ‘Yuji’ was low in nutrients and nutrient deficient. For this reason, selecting tree species that grow well in that soil environment can make the afforestation activities in that area get twice the result with half the effort.

In addition, the ‘Yuji’ forest can grow well in the above mountainous soil environment and its ecological value, and the fruit has potential economic value. The fruit can be used to extract oil. In 2020, the oil content of Yuji fruit was 24.08%. By comparing *Idesia polycarpa* ‘Yuji’ with other woody oil tree species (Table 5), it is evident that the oil content of its fruit in 2020 is subpar. It could be attributed to the particular climate conditions of that year.

**Table 5:** Oil content of *I. polycarpa* ‘Yuji’ as compared to some other woody oil tree species studied earlier.

Species	Oil content	References
*Idesia polycarpa* ‘Yuji'	24.08% (fruit), 15.13% (seeds), 27.01% (pulp)	Present study
*Acer triflorum*	28.83–48.52% (seeds)	[59]
*Camellia oleifera*	37.98% (seeds)	[60]
*Koelreuteria paniculate*	22.80% (seeds)	[61]
*Prunus armeniaca*	≥50% (seeds)	[62]
*Prinsepia utilis*	30% (fruit)	[63]
*Tilia platyphyllos*	9.10–21.70% (seeds)	[64]
*Vernicia fordii*	50.00–60.00% (seeds)	[65]
*Jatropha curcas*	30.00–40.00% (seeds)	[66]
*Cornus wilsoniana*	33.95% (fruit)	[67]
*Xanthoceras sorbifolium*	49.77–68.30% (seeds)	[68]
*Pistacia chinensis*	36.18% (fruit), 26.59% (seeds), 50.50% (pulp)	[69]

On the other hand, studies have shown that linoleic acid intake positively reduces the risk of cardiovascular disease in healthy individuals [[70], [71], [72]]. Linoleic acid is an essential metabolite of organisms and plays a vital role in growth and reproduction [[73], [74], [75]]. In this study, the linoleic acid content in fruit oil of ‘Yuji’ in 2020 reached 54.87% (Table 4). Oleic acid performs well in anti-cancer and anti-inflammatory aspects [[76], [77], [78], [79], [80]]. The oleic acid content in the Yuji fruit was 8.49%. Besides, palmitic acid content reached 22.81%. Palmitic acid can induce insulin resistance has been confirmed in most studies [[81], [82], [83], [84]]. Hence, we consider ‘Yuji’ oil to be a woody plant oil with infinite potential.

However, under this mountainous soil condition, the *Idesia polycarpa* ‘Yuji’ plantation forest grew well and showed initial results with extensive development prospects. Besides, various fatty acids were beneficial to the human body in fruit oil, which had potential economic value. Nevertheless, it is worth noting that in difficult site conditions for afforestation, the soil is only one aspect, and other factors such as temperature, length of sunlight, and precipitation must be considered. The study suggests that the other environmental factors in this area of ‘Yuji’ forest should be studied comprehensively and systematically to lay the foundation for the future use of ‘Yuji’ for afforestation in other complex sites.

## 5 Conclusions

It is significant to carry out afforestation in mountainous areas in order to study the growth condition and adaptability of the plantation forest of *Idesia polycarpa*. In this study, we investigated and analyzed the forest growth status, soil nutrient content, fruit oil content, and fatty acid composition of the 19-year-old *Idesia polycarpa* ‘Yuji’ plantation forest in the mountainous region of Henan, China. We conclude that *Idesia polycarpa* can grow normally in environments with poor soil nutrients and a high content of some fatty acids (linoleic acid 54.87%, and palmitic acid 22.81%). While playing an ecological value, it also has a potential economic value. As an emerging woody oil tree species, *Idesia polycarpa* is feasible for afforestation in mountainous areas and can be used as one of the alternative tree species. The above results may provide a further reference for afforestation in mountainous regions.

## Funding statement

This research was funded by Demonstration and promotion of fast-growing, high-yield, and high-quality cultivation technology of *Idesia polycarpa* ‘Yuji’ Maxim (GTH[2023]02).

## Data availability statement

Data will be made available on request.

## Additional information

Supplementary content related to this article has been published online at https://doi.org/10.1016/j.heliyon.2023.e19716.

## Declaration of competing interest

The authors declare that they have no known competing financial interests or personal relationships that could have appeared to influence the work reported in this paper.
