# Supplementary material for: An investigation of the growth status of 19-year-old Idesia polycarpa ‘Yuji’ plantation forest in the mountainous region of Henan, China
Source: Heliyon. 2023 Sep 1;9(9):e19716. doi: 10.1016/j.heliyon.2023.e19716 (PMC10559007; doi:10.1016/j.heliyon.2023.e19716)
Supplement: Multimedia component 1 [file mmc1.pptx]

## Slide 1
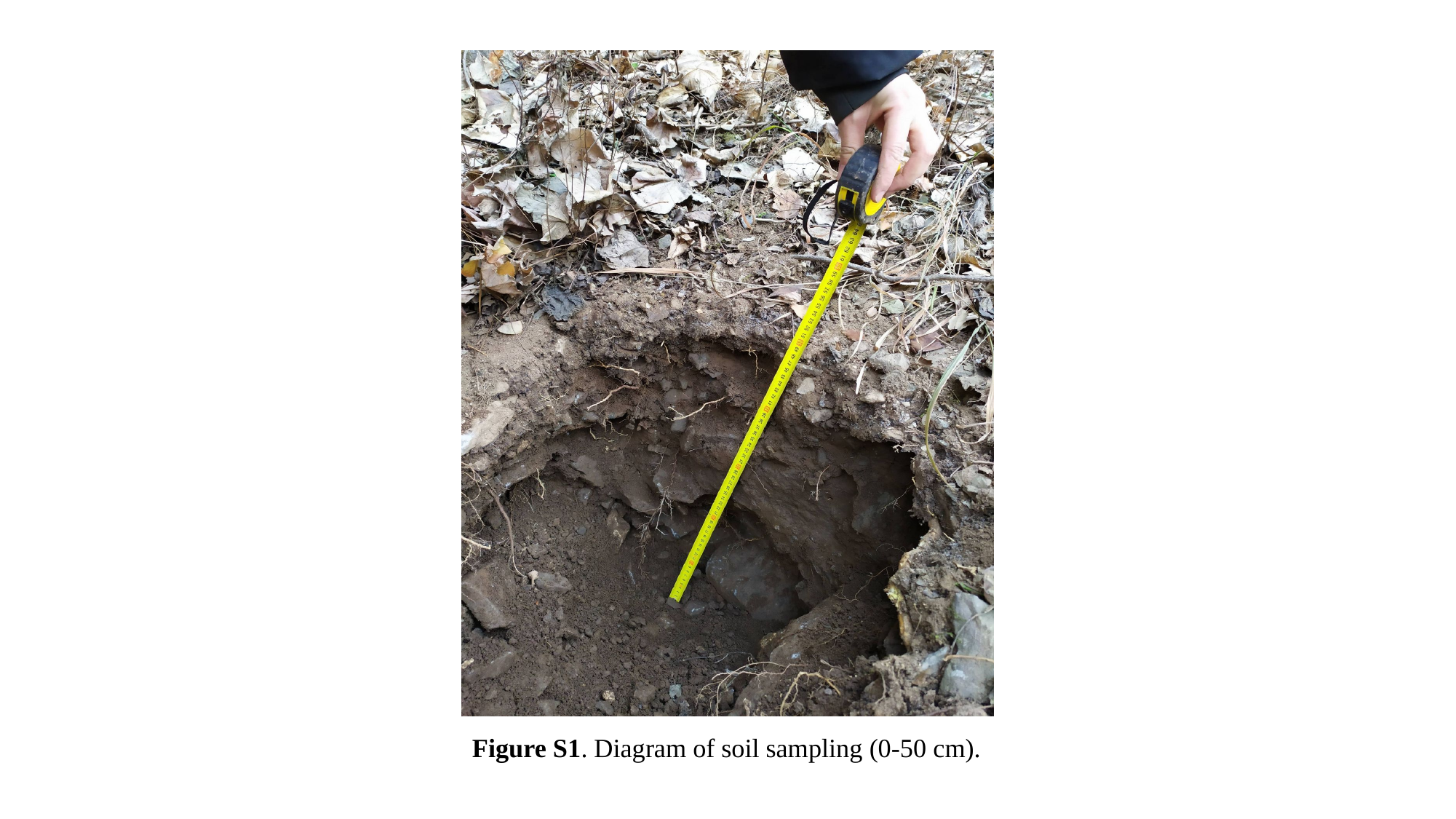

Figure S1. Diagram of soil sampling (0-50 cm).

## Slide 2
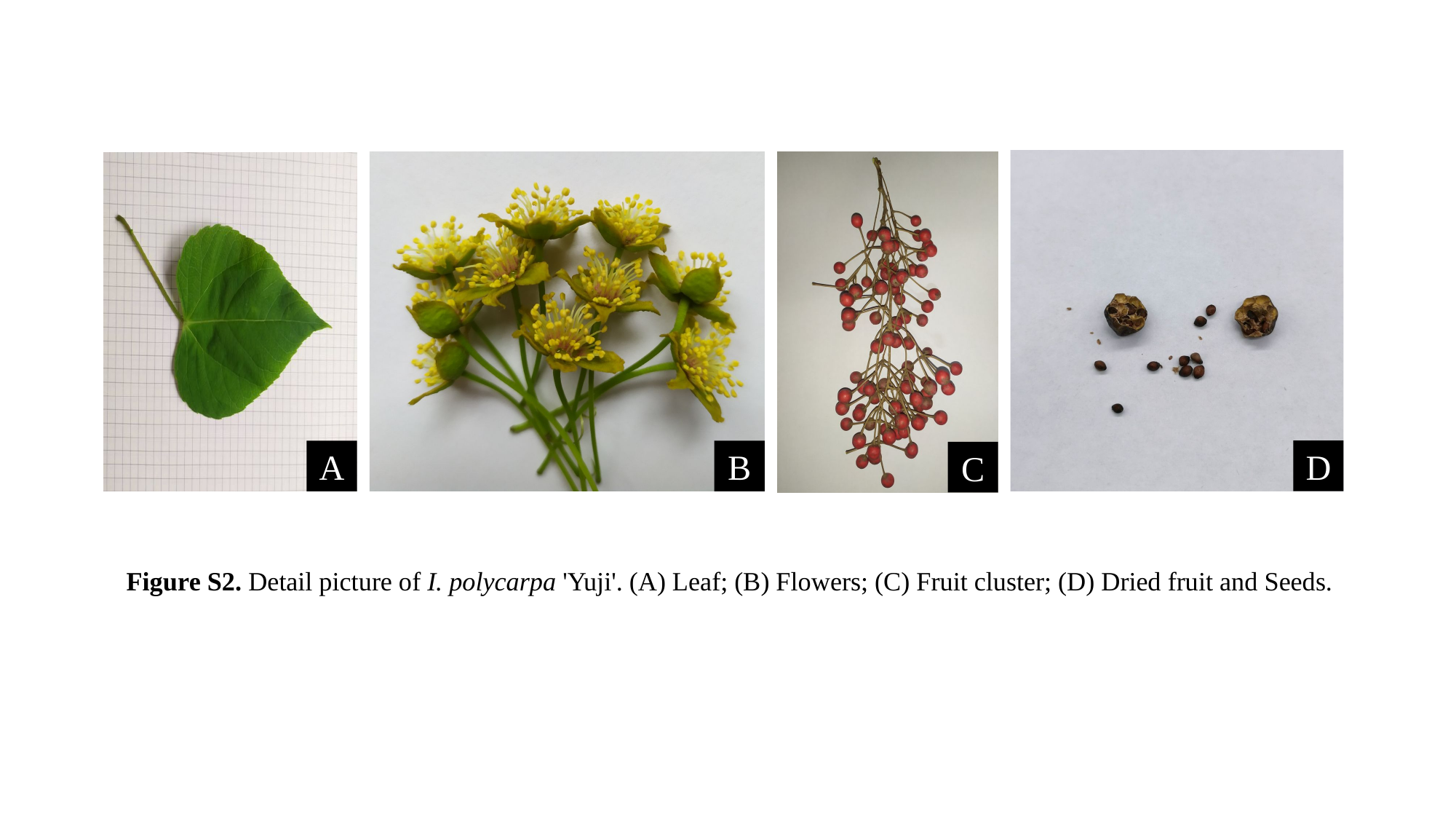

D
A
B
C
Figure S2. Detail picture of I. polycarpa 'Yuji'. (A) Leaf; (B) Flowers; (C) Fruit cluster; (D) Dried fruit and Seeds.

## Slide 3
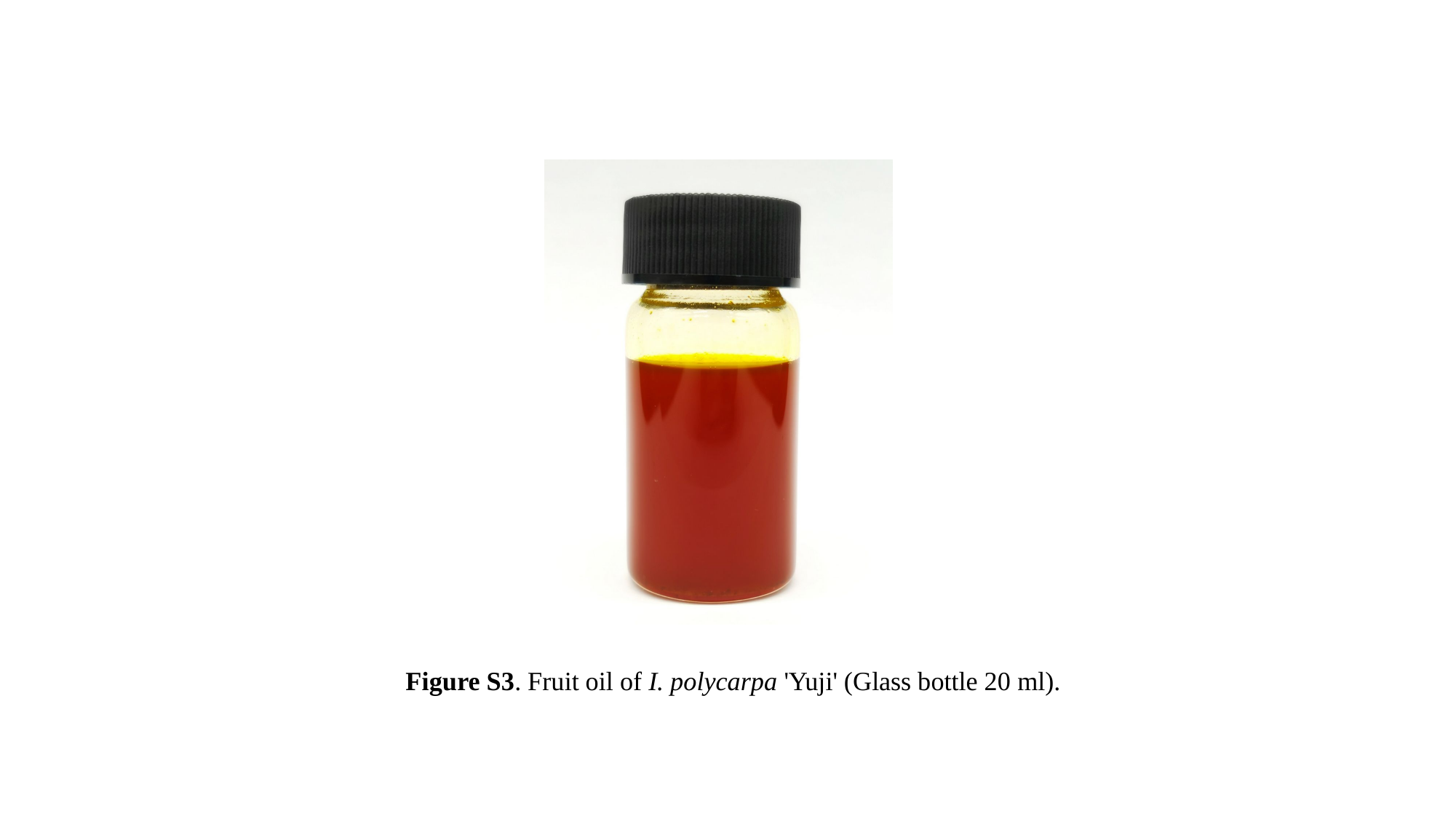

Figure S3. Fruit oil of I. polycarpa 'Yuji' (Glass bottle 20 ml).
